# The Attentional Dependence of Emotion Cognition Is Variable with the Competing Task

**DOI:** 10.3389/fnbeh.2016.00219

**Published:** 2016-11-15

**Authors:** Cheng Chen, Kaibin Jin, Yehua Li, Hongmei Yan

**Affiliations:** ^1^Key Laboratory for NeuroInformation of Ministry of Education, Center for Information in BioMedicine, University of Electronic Science and Technology of ChinaChengdu, China; ^2^Chengdu College, University of Electronic Science and Technology of ChinaChengdu, China

**Keywords:** emotion, attention, cognition, multiple resource model, dual-task

## Abstract

The relationship between emotion and attention has fascinated researchers for decades. Many previous studies have used eye-tracking, ERP, MEG, and fMRI to explore this issue but have reached different conclusions: some researchers hold that emotion cognition is an automatic process and independent of attention, while some others believed that emotion cognition is modulated by attentional resources and is a type of controlled processing. The present research aimed to investigate this controversy, and we hypothesized that the attentional dependence of emotion cognition is variable with the competing task. Eye-tracking technology and a dual-task paradigm were adopted, and subjects’ attention was manipulated to fixate at the central task to investigate whether subjects could detect the emotional faces presented in the peripheral area with a decrease or near-absence of attention. The results revealed that when the peripheral task was emotional face discrimination but the central attention-demanding task was different, subjects performed well in the peripheral task, which means that emotional information can be processed in parallel with other stimuli, and there may be a specific channel in the human brain for processing emotional information. However, when the central and peripheral tasks were both emotional face discrimination, subjects could not perform well in the peripheral task, indicating that the processing of emotional information required attentional resources and that it is a type of controlled processing. Therefore, we concluded that the attentional dependence of emotion cognition varied with the competing task.

## Introduction

The relationship between emotion and attention is an attractive cognitive topic and has fascinated researchers worldwide for decades. A variety of paradigms such as Filtering, Search, Cuing, and Multiple tasks have been used to explore the interaction of emotion and attention (Cowan, 2005; Yiend et al., 2005, Yiend, 2010; Hur et al., 2015a; Fruchtman-Steinbok et al., 2016). Important results regarding the cognitive theories and the associated brain mechanisms have been derived using multiple experimental techniques such as EEG, MEG, and fMRI (Pessoa, 2008; Arend et al., 2015). We now know that emotion and attention interact with one another and that they both affect the prioritization of information processing (Anderson and Phelps, 2001; Anderson, 2005; Most et al., 2005; Okon-Singer et al., 2013). Converging evidence has confirmed that emotional state can interfere with attention and that the latter may modulate the neural response to emotional stimuli (Yiend, 2010; Hur et al., 2015b).

However, how emotion cognition depends on attention has been the subject of long-term controversy. Some researchers hold the opinion that attention is not required for the processing of emotional stimuli. Some previous studies found that emotional visual stimuli can be processed very quickly and that this processing occurs in an automatic fashion, namely in a manner that is independent of top-down factors such as intentions and conscious awareness (Vuilleumier et al., 2001; Pasley et al., 2004; Sander et al., 2005; Vuilleumier, 2005). Vuilleumier et al. (2001) carried out a well-known study in which the attentional focus was manipulated by having subjects maintain central fixation while they were asked to compare either two faces or two houses presented eccentrically. They compared the activation in the left amygdala to fearful vs. neutral faces irrespective of the focus of attention, and they reported that differential responses in the amygdala and visual cortex were not modulated by the focus of attention, consistent with the view that the processing of emotional items does not require attention (Vuilleumier et al., 2001). Similar findings for amygdala automaticity in the processing of fear have been reported by manipulating object-based attention while maintaining a constant spatial locus of attention (Anderson et al., 2003). Norman and Tokarev (2014) recently conducted a study on the independence of face processing and also provided evidence that spatial attention does not modulate holistic face processing, even when multiple faces are present. However, many other studies in the past decade or so suggested instead that affective processing is, under many circumstances (and possibly always), under the control of attention (Pessoa et al., 2002a, 2005; Bishop et al., 2007; Straube et al., 2007; Mothes-Lasch et al., 2011). For example, Pessoa et al. (2005) found that task load was important in determining the extent of the processing of face stimuli. When the difficulty of the peripheral task was parametrically manipulated, a valence effect (i.e., fearful > neutral) was observed during conditions of low task demand but not of medium or high task demand (Pessoa et al., 2005). Mitchell et al. (2007) also hold that the processing of task-irrelevant emotional information, like neutral information, is governed by top-down processes involved in selective attention. The attentional dependence of emotion cognition on attention was also observed in studies in which centrally presented and overlapping competing stimuli were employed or in which highly aversive mutilation pictures were used (Erthal et al., 2005). Recently, Wang et al. (2016) studied the impact of perceptual load on the processing of fearful faces and also found that attentional resources are necessary for emotion processing.

Why did different studies lead to contradictory results about the attentional dependence of emotion cognition? One possible reason may be that most of the previous studies formed conclusions based on comparisons using different kinds of competing stimuli. Attentional resources are known to be limited. According to perceptual load theory, if a high perceptual load for a competing task exhausts attentional resources, then it will prevent the processing of other tasks. However, if a low perceptual load task does not exhaust attentional resources, then attention can be spared to process other tasks.

When examining subjects’ response to competing stimuli, researchers usually use the Multiple-task paradigm, which is effective and frequently used to investigate the effects of attention on different cognitive tasks. In multiple tasks, subjects must allocate their limited processing capacity to meet more than one demand when required to report two sequential or spatial targets. Attentional blink is a frequently used temporal-sequential Multiple-task method, in which subjects have to report two sequential targets in a rapid stream of stimuli (called rapid serial visual presentation; RSVP). If target 1 (T1) and target 2 (T2) are sufficiently close to each other in presentation time (T1 is presented first and then followed closely by T2, which is typically a few 100 ms later), then T2 is often missed (Raymond et al., 1992). Previous attentional blink studies showed that emotional information can either enhance or attenuate the blink, depending on whether the emotional information is identified (or in some studies merely presented) as the first or the second target (Keil and Ihssen, 2004; Most et al., 2007; Trippe et al., 2007). These results suggest that emotional information recruits extra attentional resources during these tasks, consistent with the view that attention is required for emotion perception.

Additionally, spatial Multiple-task paradigm can be used to investigate the allocation of spatial attention. For instance, using a spatial dual-task paradigm, Li et al. (2002) showed that natural scenes, e.g., animal vs. non-animal, can be categorized in the near-absence of spatial attention. Then, using the same experimental paradigm, Reddy et al. (2004) reached the similar conclusion that the attentional cost associated with the visual discrimination of face gender is also very minor.

Until now, few studies have explored the spatial attentional demands of emotion processing. In this paper, based on the characteristics of the priority and automaticity of emotional information, as well as the load theory of attention and the multiple resource model, we hypothesized that the attentional dependence of emotion cognition is variable with the competing task. If the competing task is irrelevant to emotion, both the emotional and competing tasks could be performed well. Otherwise, the performance would be impaired. A similar spatial dual-task paradigm which is employed by Li et al. (2002) was used here in this study to explore whether emotional processing could be performed well in the near-absence of spatial attention and whether emotional information could be processed in parallel with various visual tasks.

## Materials and Methods

### Subjects

Ten right-handed subjects (five male and five female), including one of the authors (Kaibin Jin), aged 19–25 years (average 21.60 ± 2.27), participated in the four experiments. All subjects had normal or corrected-to-normal vision and were provided with written informed consent prior to participation. The experimental paradigm was approved by the Ethics and Human Participants in Research Committee at the University of Electronic Sciences and Technology of China in Chengdu, China. All subjects except Kaibin Jin were blind to the purpose of the experiments.

### Emotional Face Database

The emotional stimuli consisted of 280 face pictures, which were obtained from the native Chinese Facial Affective Picture System (CFAPS; Bai et al., 2005), including 140 negative faces and 140 positive faces. Equal numbers of male and female faces were selected. All the pictures were divided into two subsets. One included 120 pictures for the single-task training experiment, and the other included 160 pictures for the dual-task testing experiment.

Upright face pictures were used in both single-task and dual-task experiments. The positive and negative pictures had significant difference in valence from each other, *p* < 0.0001 (*M* ±*SD*, positive: 6.40 ± 0.44, negative: 2.51 ± 0.33). However, since CFAPS is on a scale of 9, their average deviation from neutral on the scale had no significant difference; therefore the positive and negative stimuli were equivalent. All of the faces were shaved, with merely interior characteristics being retained. The resolution of the pictures was 260 × 300 dpi. The database of gray photographs was well matched for low-level features such as color, size, background, spatial frequency, brightness, and other physical properties.

### Experimental Setup

The tasks were performed in a sound-attenuated room that was specially designed for psychophysical experiments. The visual stimuli were presented on a 21-inch color monitor (DELL Trinitron) providing a frame frequency of 100 Hz at a spatial resolution of 1280 × 1024 pixels. The viewing distance was 57 cm, and stimuli appeared on a gray background that was adjusted to a mean luminance of approximately 22 cd/m^2^.

Although all subjects were trained psychophysical observers, we nevertheless monitored the subjects’ eye movements to guarantee no overt fixation shifting to the peripheral pictures during the dual-task experiments. Eye movements were recorded with an infrared eye tracker (EyeLink 2000, SR Research, Ltd.) and sampled at 1000 Hz. Head movements were restricted by a forehead and chin rest. The pupil of the left eye was tracked at a sampling rate of 1000 Hz and a spatial resolution of 0.1°.

### Training Procedure

The training procedure was almost similar to that of Li et al. (2002). Each experiment required a significant training period, in which the subject was instructed to finish two single-tasks. It usually took more than 10 h (∼2,500 trials for each different task) for a subject to coordinate their motor responses well enough to answer a speedy peripheral task and a central task, respectively. The central SOA (stimulus onset asynchrony, the time between the onset of the central stimulus and the onset of the central mask) started at 500 ms and then gradually decreased after each block to less than 250 ms. The procedure was terminated after the subject’s performance had stabilized, with the accuracy rate exceeding 85% and the central SOA being below 250 ms. This value was chosen to limit the possibility of attention switching during stimulus presentation. The peripheral SOA was similarly determined according to the threshold performance for each subject. It started at 500 ms and then gradually decreased to less than 200 ms. The procedure was terminated after the subject’s performance had stabilized, with the accuracy rate exceeding 82% and the peripheral SOA being below 200 ms. All tasks received the same amount of training for each subject to avoid bias for any particular task.

## Experiments

### Experiment 1: Central Letter – Peripheral Emotional Face Discrimination

The experiment consisted of three different conditions: a central task condition that is attention-demanding, a peripheral task condition in which the role of attention was investigated, and a dual-task condition in which both the central and the peripheral tasks were performed concurrently. Subjects were instructed to be as accurate as possible, and there was no limitation on reaction times because both the peripheral and the central stimuli were masked separately and successively after their corresponding SOAs, leaving no visual clues for the subjects beyond that timeframe. Subjects’ responses were collected over two sessions for each condition. Each session consisted of two blocks of 30 trials in each single-task condition and two blocks of 40 trials in the dual-task condition.

#### Central Letter Discrimination Task

Each trial started with a black dot (0.3° × 0.3°) presented 600 ± 100 ms before the onset of the first stimulus. The central stimulus was a combination of five randomly rotated Ts and Ls, each with a size of 0.8° × 0.8°, either all identical or one different from the other four, and was presented at the center of the display at one of the nine possible locations within 1.2° of fixation. After the central SOA, the letters were individually masked by an “F,” which was rotated by an angle corresponding to the “T” or “L” it replaced. The central SOA was determined individually for each subject. Individual central SOAs ranged from 200 to 250 ms, averaging 236 ms. For a given subject, the central SOA was the same for both the single-task and the dual-task conditions. Subjects were required to report whether the central letters were identical or not by pressing two keys with the left hand.

#### Peripheral Emotional Face Discrimination Task

A face picture of approximately 2.6° × 3.0° of visual angle was presented peripherally after the onset of the central fixation point. The face appeared at a random location centered at approximately 6.0° eccentricity. After the peripheral SOA, the face was masked by a pattern mask composed of random noise. Likewise, the peripheral SOA was determined individually for each subject. Individual peripheral SOAs ranged from 160 to 200 ms, averagely 180 ms. For a given subject, peripheral SOA was the same for both the single-task and the dual-task conditions. Subjects were required to report whether the emotional face was positive or negative by pressing two keys with the right hand.

#### Dual-Task Condition

**FIGURE 1:**
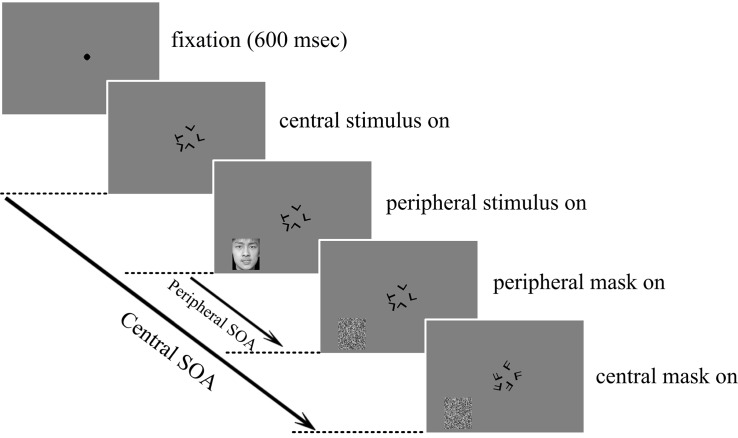
**Schematic illustration of one trial in the central letter – peripheral emotional face discrimination experiment.** Subjects were required to report whether the five central letters were the same or different and whether the peripherally presented emotion was positive or negative. Both the letters and the faces were masked individually after the corresponding stimulus onset asynchrony (SOA).

In the dual-task condition, subjects were instructed to fixate at the center and focus attention on the central task but respond to both the central and the peripheral tasks with the left and right hands, respectively, and as accurately as possible. The experimental protocol of the dual-task condition is illustrated in **Figure 1**.

### Experiment 2: Central – Peripheral Emotional Faces Discrimination

**FIGURE 2:**
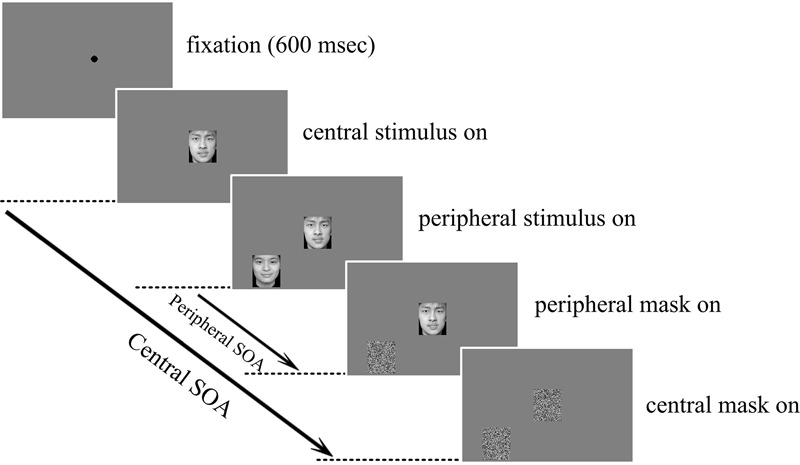
**Central – peripheral emotional faces discrimination experimental protocol.** Subjects were required to fixate at and focus attention on the central task and to respond whether the emotion was positive or negative for both the centrally and peripherally presented faces. Both the central and peripheral faces were masked individually after the corresponding SOA.

All 10 subjects from experiment 1 were asked to perform experiment 2 after 60 days. The experimental protocols for both the single and the dual-tasks in experiment 2 were similar to those in experiment 1, but the central letter stimuli in experiment 1 were replaced with emotional face pictures. The size of the central and peripheral face pictures were both 2.6° × 3.0°. Similarly, the peripheral SOA was also determined individually for each subject, ranging from 160 to 200 ms and averaging 180 ms, and the central SOA was 20 ms longer than its corresponding peripheral SOA in order to guarantee that the central stimuli were always presented earlier and masked later than the peripheral stimuli. In the dual-task condition, subjects were required to fixate at the center and focus attention on the central task but respond to both the central and the peripheral tasks with the left and right hands, respectively, and as accurately as possible. The dual-task experimental protocol is illustrated in **Figure 2**.

### Experiments 3 and 4: Central Letter – Peripheral Letter and Color Discrimination

To confirm that attention is allocated to the center of the visual field under the dual-task condition, we conducted two control experiments that were quite similar to those of Li et al. (2002). In experiment 3, the central stimulus was the same as that in experiment 1, but the peripheral stimulus was a randomly rotated letter T or L with a size of 1.5° × 1.5° and was then masked by the letter “F.” Subjects were required to report whether the peripherally presented letter was T or L by pressing two keys with the right hand. In experiment 4, the central stimulus was also the same as that in experiment 1, but the peripheral stimulus was a vertically bisected disk with red and green halves. The size of the peripheral color disk was 1.5° × 1.5° and was then masked by another disk of four quadrant swatches, with red and green alternating between quadrants. Subjects were required to report whether the bisected disk showed a pattern with red on the left and green on the right, or vice versa, by pressing two keys with the right hand. Again, before the dual-task condition, subjects finished a training period. The training procedure showed that all subjects achieved very high performance, almost 100%, for the peripheral control tasks at SOA of 150 ms. For a better match with experiments 1 and 2, we set the peripheral SOA as 200 ms for the two control experiments. Similarly, peripheral single-task and dual-task protocols were carried out separately for the two control experiments. These two control experiments aimed to confirm the fact that attention is fully allocated to the central task and that there is no decrease in the central performance under dual-task conditions compared with single-task conditions, because if the peripheral task does demand attention or an attention shift occurs, the central performance should suffer.

### Data Analysis

To exclude the possibility that the subjects might shift their fixation from the central to the peripheral stimuli or vice versa, which might contaminate the results of the dual-task experiments, off-line eye movement data were analyzed. If any saccade occurred or the gazing position of eyes deviated more than 1.5° from the fixation point during one trial, the trial was discarded. In total, less than 0.1% of trials were discarded.

**FIGURE 3:**
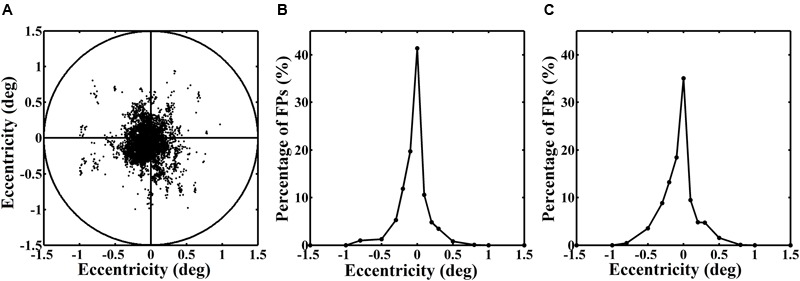
**(A)** Distribution of the real fixation positions (FPs) of the eyes in one block. **(B)** and **(C)** Distributions of the relative number (%) of the real FPs over the horizontal and vertical axes.

**Figure 3** shows an example of the real fixation positions (FPs) and their distribution in one block during one dual-task experiment. The left panel represents the distribution of the real FPs. The circle that encircles the points indicates a range of 1.5° visual angle. It is exactly within the range of the central letter stimuli. The curves in the middle and right panels illustrate the distribution of the relative number (%) of the real FPs over the horizontal and vertical axes. All of them reveal a normal distribution, with a peak at the assigned fixation point of 0° eccentricity. Thus, we ensured that the eyes always well fixated at the fixation point no matter the stimuli were presented in the central or the peripheral position.

The performances in the dual-task experiment were normalized according to each subject’s performance in the corresponding single-task experiment. A one-way ANOVA and paired *t*-tests were computed for each experiment to compare single-task and dual-task performances.

## Results

**FIGURE 4:**
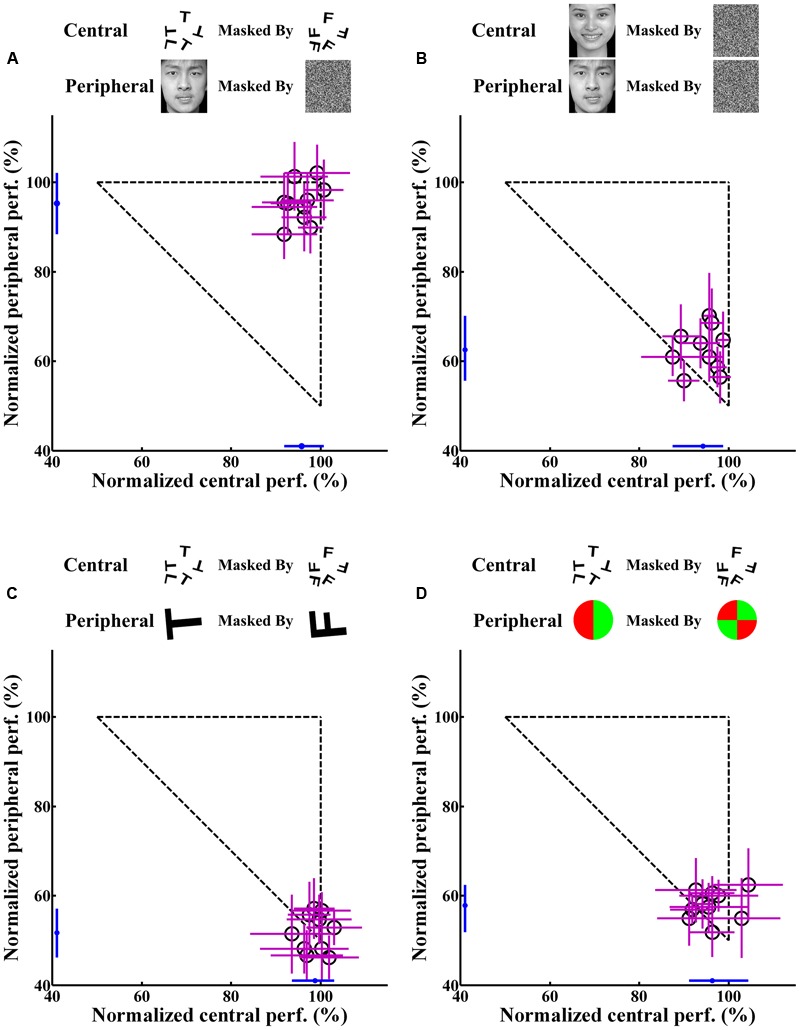
**Individual and average central and peripheral performances under the dual-task condition in the four experiments.** The horizontal axis represents the performance of the attention-demanding central task. The vertical axis represents the performance of the peripheral task. Each open circle represents the individual performance of one subject in the dual-task, and the small blue dot near the horizontal or vertical axis represents the average performance of corresponding central or peripheral task for all subjects. The accuracy rate is the normalized data. Error bars represent SD. **(A)** Central letter-peripheral emotional face discrimination task. **(B)** Central – peripheral emotional faces discrimination task. **(C)** Central – peripheral letter discrimination task. **(D)** Central letter – peripheral color disk discrimination task.

**Figure 4** shows the individual and average central and peripheral performances in the four dual-task experiments. In experiment 1, under the dual-task condition, subjects were instructed to focus attention on the central letter stimuli and to try to concurrently perform the central letter discrimination and the peripheral emotion discrimination as accurately as possible. The results showed that, for each subject, the normalized central task performance under the dual-task condition (95.81 ± 3.08%; mean ± SEM) (**Figure 4A**) showed no difference (*p* > 0.05) from its counterpart under the single-task condition (96.56 ± 2.98%). Moreover, the normalized peripheral task performance under the dual-task condition (95.36 ± 4.45%; **Figure 4A**) was also not significantly (*p* > 0.05) different from the corresponding performance under the single-task condition (95.83 ± 3.89%). Previous evidence (Li et al., 2002; Reddy et al., 2004) and our eye movement analysis clearly indicated that there was no systematic switch of attention between the two tasks and that attention was locked at the central task under the dual-task condition. Therefore, the result of experiment 1 seemed to suggest that emotion discrimination can still be performed efficiently even when attention is drawn away.

However, in experiment 2, when the central letter task was replaced by the emotion discrimination task under the dual-task condition, although the central task performance under the dual-task condition (94.25 ± 3.96%) was almost equivalent to that in the single-task condition (96.25 ± 3.52%; *p* > 0.05), the peripheral emotion discrimination performance under the dual-task condition (62.58 ± 4.89%; **Figure 4B**) decreased significantly (*p* < 0.0001) compared with that under the single-task condition (95.83 ± 3.89%).

**Figures 4C,D** show the results of control experiments 3 and 4, in which the peripheral tasks involved discriminating either a masked letter (**Figure 4C**) or a masked color disk (**Figure 4D**) in the dual-task conditions. Consistent with previous studies (Braun and Julesz, 1998; Adolphs et al., 1999; Li et al., 2002; Reddy et al., 2004), a dramatic drop was observed in the peripheral performances in both of these two control experiments. In experiments 3 and 4, the peripheral performances decreased from 99.38 ± 0.83% and 98.62 ± 1.50% in single-tasks to 51.76 ± 4.25% and 57.88 ± 3.31% in dual-tasks, respectively (*p* < 0.0001), which showed that the subjects could not do any better than random guessing during the dual-task scenarios. These results demonstrated that attention was effectively allocated to the central task and also provided evidence that extensive training did not necessarily result in an improvement in performance, given that subjects received the same amount of training in all experiments.

**FIGURE 5:**
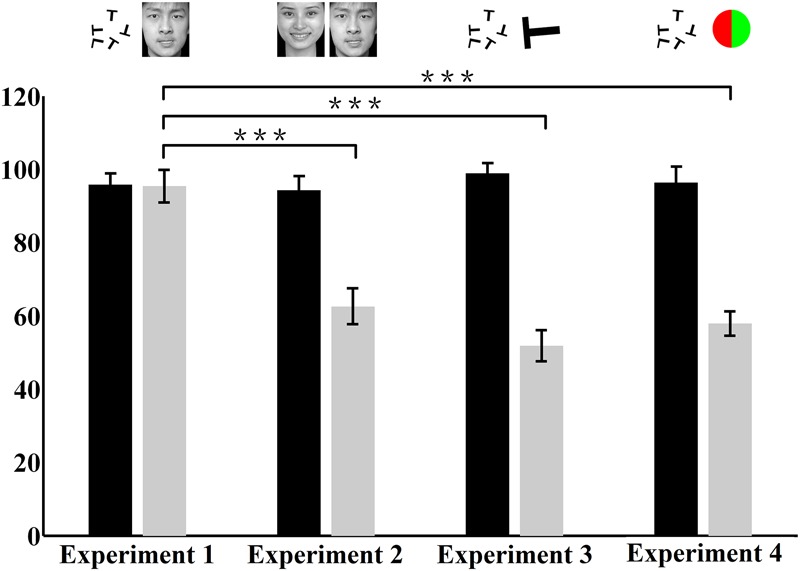
**Average central and peripheral performances in the four dual-task experiments.** The vertical axis represents the accuracy rate. The black bar represents the performance of the attention-demanding central task. The gray bar represents the performance of the peripheral task. The accuracy rate is the normalized data. The stimuli presented in the central and peripheral tasks in the four experiments are illustrated above the corresponding bars. ^∗∗∗^ *p* < 0.0001.

**Figure 5** presents the average central and peripheral performances in the four dual-task experiments. The performances in experiments 3 and 4 showed that in the peripheral area under the dual-task condition, neither the color nor the letter discrimination tasks could be performed very well, even though colors and letters are simple information. This result indicated, to a certain extent, that attention in this peripheral area did decrease or was even absent. Meanwhile, in experiments 2, subjects’ attention was manipulated in the same way as in experiments 3 and 4, and the performance on the peripheral emotion discrimination task suffered heavily. This indicated that the processing of emotion cognition was blocked in a decrease or near-absence of attention, which indicated that the processing of emotion requires attention.

However, a closer look at the result revealed that there were no significant differences in the accuracy rate in the central letter discrimination tasks among experiments 1, 3, and 4 (*p* > 0.05), but the performance in the peripheral task in experiment 1 differed significantly from those in experiments 3 and 4 (*p* < 0.0001). The central task in experiments 1, 3, and 4 was letter discrimination, identical across these experiments. Moreover, the attentional distribution in the peripheral area in experiment 1 decreased or was nearly absent, which was almost the same as in experiments 3 and 4. However, surprisingly, the accuracy rate in the peripheral task in experiment 1 (emotion discrimination) was significantly better than those in experiments 3 and 4 (letter and color discrimination). This important result indicated that emotion cognition can be performed even in an area with decrease or near-absence of attention, incongruent with the above conclusion that emotion cognition requires attention. Further analysis will be performed later to explore the causes of this contradiction.

**FIGURE 6:**
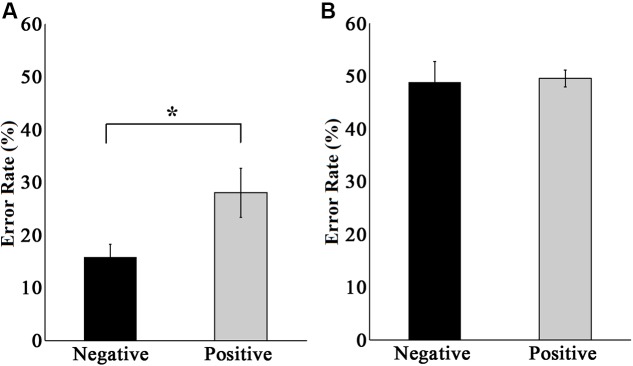
**Error rates in emotion discrimination tasks in the peripheral area in experiments 1 (A)** and 2 **(B)**. The black bar represents the error rate of all subjects when discriminating negative faces. The gray bar represents the error rate of all subjects when discriminating positive faces. The error rate is the non-normalized data. ^∗^ *p* < 0.05.

We also analyzed the error rate in peripheral emotion discrimination tasks in experiments 1 and 2, and the results are illustrated in **Figure 6**. The performance in experiment 1 (**Figure 6A**) showed that when discriminating negative faces, the non-normalized average error rate was 15.75 ± 2.48%, while when discriminating positive faces, the non-normalized average error rate was 28.00 ± 4.66%, showing a significant difference between the two situations [*t*(9) = 2.970, *p* < 0.05]. This indicated that negative emotional information was more easily cognitively processed, which is consistent with the previous reports that negative emotional information had priority in processing and could affect the attention distribution to some degree (Vuilleumier et al., 2001; Pessoa and Ungerleider, 2004; Anderson, 2005). This result provided additional evidence that under the condition in experiment 1 (central letter – peripheral emotional face discrimination task), the peripherally presented emotional faces were indeed processed by the subjects.

The performance in experiment 2 (**Figure 6B**) showed no significant difference in the error rate between discriminating negative and positive faces [*t*(9) = 0.584, *p* > 0.05]. The non-normalized average error rate of discriminating negative faces was 48.87 ± 3.88%, and that of discriminating positive faces was 49.50 ± 1.62%. These two results were essentially the same, and both were at the level of random chance. This result provided further evidence that under the condition in experiment 2 (central – peripheral emotional faces discrimination task), subjects did not switch their attention during the presentation of the stimuli, and they discriminated the peripherally presented emotional faces by randomly guessing, which means that the peripheral emotional faces were not processed at all.

## Discussion

In this study, the role of attention in emotion discrimination was studied by using a dual-task paradigm in which subjects concurrently performed a central attention-demanding task and a peripheral emotion discrimination task. The effect of attention on emotion discrimination was measured by comparing the performance on the peripheral task under the single-task condition with that under the dual-task condition. If emotion discrimination requires little or no attentional resources in any case, which means that emotional information could be processed concurrently with any other visual task, then the peripheral performance would suffer minimally in all dual-task conditions compared to the single-task condition. If the emotional task does require some attention, then the peripheral performance should be severely impaired under the dual-task conditions. If, however, the attentional dependence of emotion cognition is variable with the competing task, then the peripheral performance might be comparable when the competing task is irrelevant to emotion, and otherwise, the performance would decrease significantly.

In experiments 1 and 2, the subjects underwent the same training procedures and experimental protocols, and their attention could not switch between central and peripheral tasks in either experiment. To some extent, experiment 2 was much easier than experiment 1 because the central SOA in experiment 2 was adopted according to the peripheral emotion discrimination task and prolonged by 20 ms in order to guarantee that the central stimuli were always presented earlier and masked later than the peripheral stimuli. Nevertheless, the peripheral emotion discrimination task was performed very well concurrently with the central letter task in experiment 1, whereas it was significantly impaired when performed concurrently with a central emotion discrimination task in experiment 2. Why did this happen?

It is high time that we thoroughly examine the reason underlying the contradiction in the attentional dependence of emotion. Psychological researchers strongly agree that a wise and scientific approach would be to simultaneously adopt the load theory of attention and the multiple resource model to compare and explain the results of multi-task experiments. Load theory of attention is frequently used to explain the distribution model of attention. It proposes that the organism has a limited attentional capacity; therefore, within certain time duration, the cognitive resource that is distributed to the current task is naturally limited. This theory emphasizes that the distribution model of cognitive resource in the selective attention process is decided by the perceptual load of the current task (Lavie and Tsal, 1994; Lavie et al., 2004, 2014; Cao et al., 2014; Bobak and Langton, 2015; Tan et al., 2015). In the four experiments in the current study, there was no significant difference in the accuracy rate in the central tasks under either the dual-task or the single-task condition, demonstrating that the peripheral task did not affect the central task. This also demonstrated that the central task was a high perceptual load task that occupied most of the attentional resources of the subjects and left very little attentional resource for the peripheral area. Therefore, the peripheral stimuli cannot be processed sufficiently, and their recognition rate reached just a level of random guessing. However, in experiment 1 (central letter – peripheral emotional face discrimination task), subjects’ performance in the peripheral task was very good (**Figure 5**). This absolutely did not agree with the load theory of attention, and we therefore concluded that the load theory of attention is not suitable for explaining the results of the current study.

Thus, we turned to the multiple resource model, which is also widely acknowledged and can explain the distribution model of attention and the cognitive processing strategy (Wahn and König, 2016). This model proposes that there are multiple resource channels in the human cognitive system and that each can process certain external information in a specific way. As long as the processing demand of the stimuli does not exceed the limit of the specific processing resource, different types of information from various sensory channels can be processed simultaneously by these specific resource channels. According to the multiple resource model, the result of a dual-task condition is affected by two major factors. One is whether the two tasks require the same resource channel, and the other is whether the incoming information from a certain sensory channel exceeds the limit of the specific processing resource in that channel. If the two tasks demand different resource subsets and the limit is not reached, then the subject can present a good performance in the dual-task (Wickens, 2002, 2008).

In the current study, the central and peripheral tasks in experiment 2 were both emotional face discrimination tasks. Therefore, they required the same cognitive processing resource. Because the focal attention of the subjects was on the central task, the peripheral performance was severely impaired in the dual-task condition, with just chance-level accuracy, as shown in **Figure 4B**. However, in experiment 1, the central task was letter discrimination, while the peripheral task is emotion discrimination. These tasks were different and could therefore be processed in parallel because they consume different attention resources. Overall, our results demonstrated that the attentional dependence of emotion cognition can vary with the competing task. Recently, Kuang (2016) also discussed the attention to faces in a social context and stated that the attention in social contexts is a dynamic behavioral and cognitive process that could be flexibly employed to enhance any behaviorally salient stimuli.

However, there is a doubt about the impaired peripheral performance in experiment 2, namely, whether it was due to the perceptual competition *per se* or was simply because of the differences in the reporting costs. In experiment 2, both the central and the peripheral tasks were emotion perception tasks. It may be more challenging to memorize and report two close answers (two emotions in experiment 2) than two different category answers (letter and emotion in experiment 1). To answer this question, we did a complementary experiment and recruited another 10 right-handed subjects (three male and seven female), aged 22–33 years (23.70 ± 3.36). The experimental protocols in the additional experiment were exactly the same with those in experiment 2, but the central task was face gender discrimination.

According to the previous study conducted by Reddy et al. (2004), subjects can response to face gender very quickly within about 145 ms when the stimuli were presented in the peripheral area. Therefore, we changed the contrast of the centrally presented face stimuli to increase the task difficulty so as to guarantee that subjects fixated at and locked their focal attention on the central task. The results showed that the normalized central task performance under the dual-task condition (94.44 ± 1.52%) was almost equivalent to its counterpart in the single-task condition (94.64 ± 1.18%; *p* > 0.919), but the normalized peripheral task performance under the dual-task condition (70.45 ± 2.47%) decreased significantly compared with the corresponding performance under the single-task condition (100.02 ± 1.45%; *p* < 0.0001).

In the additional experiment, although reporting gender and emotion is easier than reporting two emotions, the peripheral performance was still heavily impaired and was not improved substantially compared with that in experiment 2. Therefore, we hold the view that the impaired peripheral performance in experiment 2 was not caused by the reporting cost, but was due to the perceptual task *per se*. Actually, it has already proved by Li et al. (2002), and Rousselet et al. (2002) by using event related potentials (ERP), that it is not necessarily the complexity of the visual discrimination task that determines whether it can be performed in the near-absence of attention; the type of stimuli also plays an important role in determining the attentional demands of the task. Our results were consistent with these previous studies.

Our results in experiment 1 suggested that emotion cognition can be performed well even in an area with decrease or near-absence of attention when the competing task is irrelevant to emotion, which implied that emotion can be processed in parallel with some other kind of information. Some neurophysiological studies about emotion cognition reported that the amygdala plays a crucial role in emotion processing and contains two different components when processing emotional information. One is an early, rapid, automatic processing component that is independent of the cognitive load and attentional resource (Sander et al., 2005). The other is a late, top-down, attention-controlled component (Pessoa et al., 2002b; Bishop et al., 2007; Mothes-Lasch et al., 2011; Wang et al., 2014). This dual-path model postulates that the amygdala provides two pathways in emotion processing, namely the subcortical and cortical pathways. The subcortical pathway can roughly but rapidly process simple emotional information, such as the fearful faces that suddenly appear in the low-sensitivity peripheral vision area, fearful pictures that are vague and low in spatial resolution, and similar stimuli. This pathway responds after only approximately 120 ms after the onset of the stimulus, which is very quickly and far earlier than the elaborate processing of faces by the visual cortex, as well as earlier than any attention modulation component (Pourtois et al., 2005; Eimer and Holmes, 2007). The cortical pathway can address emotional information elaborately but slowly, at approximately 170 ms after the stimulus onset. The two pathways and their corresponding automaticity and modulation components in emotion processing interact with each other. On the one hand, this integration mechanism reflects the capacity of humans to rapidly detect and respond to danger and threat; on the other hand, it indicates that advanced cognitive function can accomplish further processing of emotion and affect behavioral decisions. According to this report, emotion cognition is variable to some extent, and we can consider it a type of controlled processing.

## Conclusion and Limitations

In summary, the current study adopted a dual-task paradigm to investigate the relationship between emotion cognition and attention, as well as the possible neuromechanism. After all of the detailed analyses and explanations, it is necessary to clearly state our results as follows: First, emotion cognition required very little attention when the competing task was irrelevant to emotion or emotion related types. Second, when both the central and peripheral tasks were discrimination of emotional faces, the subjects did not perform well in the peripheral task, showing that similar attentional resources affected emotion cognition and it was the perceptual task *per se* of the competing task, instead of the reporting cost, that impaired the performance in emotion cognition. Overall, the attentional dependence of emotion cognition is variable with the competing task and is a type of controlled processing. These results indicated that emotional information can be processed in parallel with other stimuli and that there may be a specific channel in the human brain to process emotional information.

Since this is a behavioral study, and it is difficult to objectively quantify the research conditions and results, it inevitably yielded limitations and suggested possible future research. Firstly, we hold the view that emotion is a specific kind of stimulus, which is different from and can be processed in parallel with certain other stimuli such as letters and colors. However, the stimuli used in this study were quite limited, so it would be a good idea to employ more types of stimuli in the future, such as animals, orientations, and shapes. Moreover, although the eye movements were recorded in this study, the brain activity was not detected. Therefore, future studies may use ERP, fMRI, and other approaches to investigate the brain activity under the dual-task condition and thus further verify whether there is a specific attention channel for emotional information. In addition, only visual stimuli were used in this study. Because emotional information can also be perceived via other sensory channels, such as audio, touch, and smell, and in order to verify the existence of the specific emotional channel, future studies may also employ various forms of stimuli and testify whether emotional information coming from other sensory channels can be processed in parallel with other types of information.

## Author Contributions

Conceived and designed the experiments: HY. Performed the experiments: KJ, YL, and CC. Analyzed the data: KJ, CC, and YL. Wrote the paper: CC and HY.

## Conflict of Interest Statement

The authors declare that the research was conducted in the absence of any commercial or financial relationships that could be construed as a potential conflict of interest.

## References

[B1] Adolphs R. Tranel D. Hamann S. Young A. W. Calder A. J. Phelps E. A. (1999). Recognition of facial emotion in nine individuals with bilateral amygdala damage. *Neuropsychologia* 37 1111–1117. 10.1016/S0028-3932(99)0039-1 10509833

[B2] Anderson A. K. (2005). Affective influences on the attentional dynamics supporting awareness. *J. Exp. Psychol. Gen.* 134 258–281. 10.1037/0096-3445.134.2.258 15869349

[B3] Anderson A. K. Christoff K. Panitz D. De Rosa E. Gabrieli J. D. (2003). Neural correlates of the automatic processing of threat facial signals. *J. Neurosci.* 23 5627–5633. 12843265 10.1523/JNEUROSCI.23-13-05627.2003 PMC6741280

[B4] Anderson A. K. Phelps E. A. (2001). Lesions of the human amygdala impair enhanced perception of emotionally salient events. *Nature* 411 305–309. 10.1038/35077083 11357132

[B5] Arend I. Henik A. Okon-Singer H. (2015). Dissociating emotion and attention functions in the pulvinar nucleus of the thalamus. *Neuropsychology* 29 191–196. 10.1037/neu0000139 25180982

[B6] Bai L. Ma H. Huang Y. Luo Y. (2005). The development of native chinese affective picture system—a pretest in 46 college students. *Chin. Ment. Health J.* 19 719–722.

[B7] Bishop S. J. Jenkins R. Lawrence A. D. (2007). Neural processing of fearful faces: effects of anxiety are gated by perceptual capacity limitations. *Cereb. Cortex* 17 1595–1603. 10.1093/cercor/bhl070 16956980

[B8] Bobak A. K. Langton S. R. H. (2015). Working memory load disrupts gaze-cued orienting of attention. *Front. Psychol.* 6:1258 10.3389/fpsyg.2015.01258 PMC4547003 26379587

[B9] Braun J. Julesz B. (1998). Withdrawing attention at little or no cost: detection and discrimination tasks. *Percept. Psychophys.* 60 1–23. 10.3758/BF03211915 9503909

[B10] Cao H. Jin K. Li C. Yan H. (2014). Attentional blink is hierarchically modulated by phonological, morphological, semantic and lexical connections between two Chinese characters. *PLoS ONE* 9:e104626 10.1371/journal.pone.0104626 PMC4125286 25101959

[B11] Cowan N. (2005). “Selective attention tasks in cognitive research,” in *Cognitive Methods in Clinical Research* eds Wenzel A. Rubin D. C. (Washington, DC: American Psychological Association) 73–96.

[B12] Eimer M. Holmes A. (2007). Event-related brain potential correlates of emotional face processing. *Neuropsychologia* 45 15–31. 10.1016/j.neuropsychologia.2006.04.022 16797614 PMC2383989

[B13] Erthal F. S. De Oliveira L. Mocaiber I. Pereira M. G. Machado-Pinheiro W. Volchan E. (2005). Load-dependent modulation of affective picture processing. *Cogn. Affect. Behav. Neurosci.* 5 388–395. 10.3758/CABN.5.4.388 16541809

[B14] Fruchtman-Steinbok T. Salzer Y. Henik A. Cohen N. (2016). The interaction between emotion and executive control: comparison between visual, auditory, and tactile modalities. *Q. J. Exp. Psychol.* 10.1080/17470218.2016.1199717 [Epub ahead of print]. 27295071

[B15] Hur J. Iordan A. D. Berenbaum H. Dolcos F. (2015a). Emotion-attention interactions in fear conditioning: moderation by executive load, neuroticism, and awareness. *Biol. Psychol.* 10.1016/j.biopsycho.2015.10.007 [Epub ahead of print]. 26522991

[B16] Hur J. Miller G. A. McDavitt J. R. Spielberg J. M. Crocker L. D. Infantolino Z. P. (2015b). Interactive effects of trait and state affect on top-down control of attention. *Soc. Cogn. Affect. Neurosci.* 10 1128–1136. 10.1093/scan/nsu163 25556211 PMC4526484

[B17] Keil A. Ihssen N. (2004). Identification facilitation for emotionally arousing verbs during the attentional blink. *Emotion* 4 23–35. 10.1037/1528-3542.4.1.23 15053724

[B18] Kuang S. (2016). Two polarities of attention in social contexts: from attending-to-others to attending-to-self. *Front. Psychol.* 7:63 10.3389/fpsyg.2016.00063 PMC4734343 26869965

[B19] Lavie N. Beck D. M. Konstantinou N. (2014). Blinded by the load: attention, awareness and the role of perceptual load. *Philos. Trans. R. Soc. B* 369:20130205 10.1098/rstb.2013.0205 PMC3965161 24639578

[B20] Lavie N. Hirst A. de Fockert J. W. Viding E. (2004). Load theory of selective attention and cognitive control. *J. Exp. Psychol. Gen.* 133 339–354. 10.1037/0096-3445.133.3.339 15355143

[B21] Lavie N. Tsal Y. (1994). Perceptual load as a major determinant of the locus of selection in visual attention. *Percept. Psychophys.* 56 183–197. 10.3758/BF03213897 7971119

[B22] Li F. VanRullen R. Koch C. Perona P. (2002). Rapid natural scene categorization in the near absence of attention. *Proc. Natl. Acad. Sci. U.S.A.* 99 9596–9601. 10.1073/pnas.092277599 12077298 PMC123186

[B23] Mitchell D. G. V. Nakic M. Fridberg D. Kamel N. Pine D. S. Blair R. J. (2007). The impact of processing load on emotion. *Neuroimage* 34 1299–1309. 10.1016/j.neuroimage.2006.10.012 17161627 PMC1909754

[B24] Most S. B. Chun M. M. Widders D. M. (2005). Attentional rubbernecking: cognitive control and personality in emotion-induced blindness. *Psychon. Bull. Rev.* 12 654–661. 10.3758/BF03196754 16447378

[B25] Most S. B. Smith S. D. Cooter A. B. Levy B. N. Zald D. H. (2007). The naked truth: positive, arousing distractors impair rapid target perception. *Cogn. Emot.* 21 964–981. 10.1080/02699930600959340

[B26] Mothes-Lasch M. Mentzel H.-J. Miltner W. H. R. Straube T. (2011). Visual attention modulates brain activation to angry voices. *J. Neurosci.* 31 9594–9598. 10.1523/JNEUROSCI.6665-10.2011 21715624 PMC6623173

[B27] Norman L. J. Tokarev A. (2014). Spatial attention does not modulate holistic face processing, even when multiple faces are present. *Perception* 43 1341–1352. 10.1068/p7848 25669051

[B28] Okon-Singer H. Lichtenstein-Vidne L. Cohen N. (2013). Dynamic modulation of emotional processing. *Biol. Psychol.* 92 480–491. 10.1016/j.biopsycho.2012.05.010 22676964

[B29] Pasley B. N. Mayes L. C. Schultz R. T. (2004). Subcortical discrimination of unperceived objects during binocular rivalry. *Neuron* 42 163–172. 10.1016/S0896-6273(04)00155-2 15066273

[B30] Pessoa L. (2008). On the relationship between emotion and cognition. *Nat. Rev. Neurosci.* 9 148–158. 10.1038/nrn2317 18209732

[B31] Pessoa L. Kastner S. Ungerleider L. G. (2002a). Attentional control of the processing of neutral and emotional stimuli. *Cogn. Brain Res.* 15 31–45. 10.1016/S0926-6410(02)00214-8 12433381

[B32] Pessoa L. McKenna M. Gutierrez E. Ungerleider L. G. (2002b). Neural processing of emotional faces requires attention. *Proc. Natl. Acad. Sci. U.S.A.* 99 11458–11463. 10.1073/pnas.172403899 12177449 PMC123278

[B33] Pessoa L. Padmala S. Morland T. (2005). Fate of unattended fearful faces in the amygdala is determined by both attentional resources and cognitive modulation. *Neuroimage* 28 249–255. 10.1016/j.neuroimage.2005.05.048 15993624 PMC2427145

[B34] Pessoa L. Ungerleider L. G. (2004). Neuroimaging studies of attention and the processing of emotion-laden stimuli. *Prog. Brain Res.* 144 171–182. 14650848 10.1016/S0079-6123(03)14412-3

[B35] Pourtois G. Dan E. S. Grandjean D. Sander D. Vuilleumier P. (2005). Enhanced extrastriate visual response to bandpass spatial frequency filtered fearful faces: time course and topographic evoked-potentials mapping. *Hum. Brain Mapp.* 26 65–79. 10.1002/hbm.20130 15954123 PMC6871777

[B36] Raymond J. E. Shapiro K. L. Arnell K. M. (1992). Temporary suppression of visual processing in an RSVP task: an attentional blink? *J. Exp. Psychol. Hum. Percept. Perform.* 18 849–860. 10.1037/0096-1523.18.3.849 1500880

[B37] Reddy L. Wilken P. Koch C. (2004). Face-gender discrimination is possible in the near-absence of attention. *J. Vis.* 4 106–117. 10.1167/4.2.4 15005651

[B38] Rousselet G. A. Fabre-Thorpe M. Thorpe S. J. (2002). Parallel processing in high-level categorization of natural images. *Nat. Neurosci.* 5 629–630. 10.1038/nn866 12032544

[B39] Sander D. Grandjean D. Pourtois G. Schwartz S. Seghier M. L. Scherer K. R. (2005). Emotion and attention interactions in social cognition: brain regions involved in processing anger prosody. *Neuroimage* 28 848–858. 10.1016/j.neuroimage.2005.06.023 16055351

[B40] Straube T. Weiss T. Mentzel H. J. Miltner W. H. (2007). Time course of amygdala activation during aversive conditioning depends on attention. *Neuroimage* 34 462–469. 10.1016/j.neuroimage.2006.08.021 17070072

[B41] Tan J. Zhao Y. Wang L. Tian X. Cui Y. Yang Q. (2015). The competitive influence of perceptual load and working memory guidance on selective attention. *PLoS ONE* 10:e0129533 10.1371/journal.pone.0129533 PMC4476695 26098079

[B42] Trippe R. H. Hewig J. Heydel C. Hecht H. Miltner W. H. (2007). Attentional blink to emotional and threatening pictures in spider phobics: electrophysiology and behavior. *Brain Res.* 1148 149–160. 10.1016/j.brainres.2007.02.035 17367765

[B43] Vuilleumier P. (2005). How brains beware: neural mechanisms of emotional attention. *Trends Cogn. Sci.* 9 585–594. 10.1016/j.tics.2005.10.011 16289871

[B44] Vuilleumier P. Armony J. L. Driver J. Dolan R. J. (2001). Effects of attention and emotion on face processing in the human brain: an event-related fMRI study. *Neuron* 30 829–841. 10.1016/S0896-6273(01)00328-2 11430815

[B45] Wahn B. König P. (2016). Attentional resource allocation in visuotactile processing depends on the task, but optimal visuotactile integration does not depend on attentional resources. *Front. Integr. Neurosci.* 10:13 10.3389/fnint.2016.00013 PMC4781873 27013994

[B46] Wang L. Feng C. Mai X. Jia L. Zhu X. Luo W. (2016). The impact of perceptual load on the non-conscious processing of fearful faces. *PLoS ONE* 11:e0154914 10.1371/journal.pone.0154914 PMC4858266 27149273

[B47] Wang S. Tudusciuc O. Mamelak A. N. Ross I. B. Adolphs R. Rutishauser U. (2014). Neurons in the human amygdala selective for perceived emotion. *Proc. Natl. Acad. Sci. U.S.A.* 111 E3110–E3119. 10.1073/pnas.1323342111 24982200 PMC4121793

[B48] Wickens C. D. (2002). Multiple resources and performance prediction. *Theor. Issues Ergon. Sci.* 3 159–177. 10.1080/14639220210123806

[B49] Wickens C. D. (2008). Multiple resources and mental workload. *Hum. Fact. J. Hum. Fact. Ergon. Soc.* 50 449–455. 10.1518/001872008X288394 18689052

[B50] Yiend J. (2010). The effects of emotion on attention: a review of attentional processing of emotional information. *Cogn. Emot.* 24 3–47. 10.1080/02699930903205698

[B51] Yiend J. Mathews A. Cowan N. (2005). “Selective attention in clinical and cognitive research,” in *Cognitive Methods in Clinical Research* eds Wenzel A. Rubin D. C. (Washington, DC: American Psychological Association) 65–71.

